# Alleviation of the effects of saline-alkaline stress on maize seedlings by regulation of active oxygen metabolism by *Trichoderma asperellum*

**DOI:** 10.1371/journal.pone.0179617

**Published:** 2017-06-27

**Authors:** Jian Fu, Zhihua Liu, Zuotong Li, Yufeng Wang, Kejun Yang

**Affiliations:** 1 College of Agronomy, Heilongjiang Bayi Agricultural University/Key Laboratory of Crop Germplasm Improvement and Cultivation in Cold Regions of Education Department, Daqing, People’s Republic of China; 2 School of Forestry, Northeast Forestry University, Harbin, People’s Republic of China; Universita degli Studi di Pisa, ITALY

## Abstract

This study investigated the influence of *Trichoderma asperellum* on active oxygen production in maize seedlings under saline–alkaline stress conditions. Two maize cultivars were tested: ‘Jiangyu 417’ (‘JY417’), which can tolerate saline–alkaline stress; and, ‘Xianyu 335’ (‘XY335’), which is sensitive to saline–alkaline stress. The seedlings were grown on natural saline–alkaline soil (pH 9.30) in plastic pots. To each liter of saline–alkaline soil, 200 mL of *T*. *asperellum* spore suspension was applied; three fungal suspensions were used, namely, 1 × 10^3^, 1 × 10^6^, and 1 × 10^9^ spores/L. A control with only the vehicle applied was also established, along with a second control in which untreated meadow soil (pH 8.23) was used. Root and leaf samples were collected when the seedlings had three heart-shaped leaves and the fourth was in the developmental phase. Physical and biochemical parameters related to oxidation resistance were assessed. The results indicated that the ‘JY417’ and ‘XY335’ seedlings showed different degrees of oxidative damage and differences in their antioxidant defense systems under saline–alkaline stress. As the spore density of the fungal suspension increased, the K^+^ and Ca^2+^ contents in the seedlings increased, but Na^+^ content decreased. Moreover, fungal treatment promoted the synthesis or accumulation of osmolytes, which enhanced the water absorbing capacity of the cells, increased antioxidant enzyme activities, enhanced the content of non-enzyme antioxidants, and reduced the accumulation of reactive oxygen species. Fungal treatment alleviated oxidative damage caused by the saline–alkaline stress in roots and leaves of the seedlings. The application of *T*. *asperellum* overcame the inhibitory effect of saline–alkaline soil stress on the growth of maize seedlings. In the present experiment, application with 1 × 10^9^ spores/L gave the optimal results.

## Introduction

The Songnen Plain, located in the southwest of Heilongjiang Province, is one of the leading grain-producing regions of China. An area of approximately 5,821,000 hm^2^ is planted with maize (*Zea mays* L.), and this area is greater than that used for any other crop in Heilongjiang Province. Maize yields of about 35,404,000 t/year are achieved. The high and stable yield of maize in this region is vital to the economic development of China. However, the Songnen Plain is one of the three major regions in the world with a large proportion of soda saline–alkaline land. The saline–alkaline land on the plain occupies an area of 2.882 × 10^6^ hm^2^, and severely restricts the grain yield in this area. Maize shows medium sensitivity to salinity and alkalinity and its tolerance to these conditions varies considerably among cultivars. The yield of maize on saline–alkaline soil can be reduced by 20 to 46%. Under saline–alkaline stress, substantial amounts of reactive oxygen species (ROS) are produced in plants, leading to gradual peroxidation of lipids and changes in the activities of antioxidant enzymes [1]. Continuous oxidative stress elicits stress-responses in the plant and triggers enzymatic and non-enzymatic antioxidant defense systems to help withstand the oxidative stress [2–5]. Under saline–alkaline stress, maize plants produce excessive reactive oxygen species (ROS), leading to peroxidation of the constituents of the cell membrane, which changes the activities of intracellular enzymes that scavenge active oxygen species and increases the content of thiobarbituric acid reacting substances (TBARS) [6, 7]. During evolution, plants have developed mechanisms to reduce ROS damage. These mechanisms fall into two categories, namely enzymatic and non-enzymatic detoxification systems, and are responsible for coordinating the removal of superfluous ROS from plant cells and are, thus, significant for the maintenance of the intracellular redox equilibrium [8]. Moreover, ions and osmolytes accumulate in the cells to reduce the osmotic potential and water potential of cells, and make it feasible for them to continue absorbing water so that normal plant growth can be maintained under adverse conditions [9].

*Trichoderma* spp. are soil-living fungi that associate with plant roots and have important bio-control and plant growth-promoting activities. In plants growing under saline–alkaline stress, *Trichoderma* treatments boost seed germination, alter cell osmolarity, modify expression of oxidative stress-related genes, and thereby improve tolerance to the stress [10–15]. The activities of antioxidant enzymes in plants increase to some extent after treatment with *Trichoderma*, whereas the level of TBARS decreases; these changes reduce physiological damage to the plants under saline–alkaline stress [16][16]. *Trichoderma* treatments also increase the levels of Ca^2+^ and K^+^ in plants and reduce the concentration of Na^+^, thus alleviating the impact of salt damage on the plants [17, 18]. The importance of *Trichoderma* treatments to reduce abiotic stress effects and improve yield has been studied in a range of vegetables, maize, wheat, and soybean [13, 19–23]. However, these studies have mostly used manual simulation test methods. It is unclear how these test results apply to natural saline-alkaline soils in cold regions as these conditions are complex and hard to simulate. Therefore, the effect of *Trichoderma* treatment on inducing saline–alkaline tolerance in maize under natural conditions is uncertain.

In the present study, we analyzed the effect of “Dedicated *Trichoderma* of maize” treatment on the alleviation of stress in maize seedlings grown on saline–alkaline soil. Our results here provide new insights into the mechanisms involved in the maintenance of osmotic pressure and for the improvement in ROS scavenging following *Trichoderma* treatment, and lay the foundation to understand the effects of *T*. *asperellum* in stimulating stress responses in maize seedlings and for determining the optimum concentration of *T*. *asperellum* for obtaining this effect.

## Materials and methods

### Experiment materials and design

The experiment was conducted between 2015 and 2016 at the Key Laboratory of Crop Germplasm Improvement and Cultivation in Cold Region, Daqing (125°03ʹE, 46°58ʹN, altitude 150 m), Heilongjiang Province, China. Two maize cultivars with different performances on saline–alkaline soils were used in this study: ‘Jiangyu 417’ (‘JY417’) is a highly salt-tolerant cultivar; and, ‘Xianyu 335’ (‘XY335’) is a salt-sensitive cultivar. Unbroken maize seeds (germination rate >90%) of uniform size were selected and surface sterilized with 10% sodium hypochlorite solution for 10 min; the seeds were then rinsed with sterile distilled water, air dried, and placed in an incubator at 25°C in the dark for two days to promote germination. Seeds with the same sprout lengths were selected and transplanted into plastic pots (12-cm wide and 11-cm height). Soil (pH 9.30) procured from a typical saline–alkaline area of Daqing was used for potting. The soil was air dried, thoroughly mixed, and passed through a 2-mm sieve, and each plastic pot was filled with 700 g of the soil. Each treatment had ten replicate pots, with each pot containing five seedlings. The experiments were repeated five times. *Trichoderma asperellum* (in a formulation named “Dedicated *Trichoderma* of Maize” and produced by the *Trichoderma* Research Team of the Forest Protection Project in the Academy of Forestry of Northeast Forestry University) was used to treat the seedlings. A high concentration of *T*. *asperellum* spore suspension (1 × 10^9^ spores/L) was prepared and diluted as appropriate so that the following treatments were obtained by adding 200 mL of the suspension to each liter of soil: 1 × 10^3^ (A1); 1 × 10^6^ (A2); and 1 × 10^9^ (A3) spores/L. A control given 200 mL of vehicle without spores was prepared (Con1); a second control using meadow soil (pH 8.23) was also established (Con2). The experiment was conducted in a semi-controlled growth chamber adjusted to 25/20°C day/night temperatures, and a 16-h photoperiod with photosynthetic photon flux density (PPFD) of 1000 μmol·m^-2^·s^-1^ and ~60–80% relative humidity. Tap water was supplied daily to maintain soil moisture. The basic physicochemical properties of the soil are listed in Table 1.

**Table 1:** Basic physicochemical properties of the experimental soil.

Treatment	pH	Total nitrogen (g/kg)	Total phosphorus (g/kg)	Alkali-hydrolyzable nitrogen(mg/kg)	Rapid available phosphorus (mg/kg)	Rapidly available potassium(mg/kg)	Organic matter(g/kg)
Saline-alkal Soil Con1	9.30	1.04	0.35	101.76	5.61	78.32	15.12
Meadow Soil Con2	8.23	2.06	0.44	123.12	11.45	105.78	30.24

### Plant sample collection and pretreatment

Photographs of the maize seedlings were taken when they reached the three heart-shaped leaf stage and the fourth leaf was developing. The seedlings were removed from the soil and separated into above ground tissues and roots. The midribs of the leaves were removed and the leaves were frozen in liquid nitrogen. The roots were washed under running water and then rinsed three times in distilled water. The roots were dried and frozen in liquid nitrogen. Leaves and roots were stored at -80°C until used for antioxidant enzyme activity assays.

### Determination of ion content

Ground dry plant samples (100 mg each) were digested with 2 mL sulfuric peroxide digestion mixture until a clear and almost colorless solution was obtained. After digestion, the volume of each sample was made up to 100 mL using distilled deionized water. Na^+^ and K^+^ contents were determined by flame photometry. Ca^2+^ content was determined using a slightly modified version of the technique described by Zarcinas et al. [24].

### Determination of oxidation parameters

Leaves from 27-day-old ‘JY417’ and ‘XY335’ seedlings subjected to saline–alkaline stress were stained with nitroblue tetrazolium (NBT) following the procedure described by Zhang et al. [25].

Hydrogen peroxide (H_2_O_2_) contents of leaf and root tissues (500 mg) were determined. Both tissues were homogenized in an ice bath with 5 mL 0.1% (w/v) trichloroacetic acid (TCA). The homogenate was centrifuged at 12,000 × *g* for 15 min and 0.5 mL of the supernatant was added to 0.5 mL 10 mM potassium phosphate buffer (pH 7.0) and 1 mL 1 M KI. Absorbance was read at 390 nm. The content of H_2_O_2_ was determined using a standard curve [26].

Determination of TBARS content was performed as described by Hodges et al. [27]. Frozen leaf and root tissues (0.5 g) were homogenized in 10 mL 0.1% TCA (w/v) in an ice bath, filtered and centrifuged at 28,710 × *g* and 4°C for 10 min. Two mL of extract were mixed with an equal volume of either (i) 20% (w/v) TCA solution or (ii) 20% TCA solution containing 0.5% (w/v) thiobarbituric acid. The mixtures were incubated in a hot bath (95°C) for 30 min and centrifuged at 10,000 × *g* and 4°C for 10 min. Absorbance was read at 450 nm, 532 nm, and 600 nm [27].

Superoxide anions (O_2_^·-^) content was determined using a slightly modified version of the protocol described by Elstner and Heupel [28].

### Determination of soluble osmotic adjustment substance

Soluble sugars were extracted and analyzed according to Ci et al. [29].

Proline content was determined following the method of Bates et al. [30]. Leaf and root tissue (0.2 g) was homogenized in 4 mL sulphosalicylic acid (3%) and centrifuged at 10,000 × *g* for 30 min. Two mL of the supernatant were placed in a test tube and 2 mL glacial acetic acid and 2 mL ninhydrin reagent were added. The reaction mixture was boiled in a water bath at 100°C for 30 min. After cooling, 4 mL of toluene were added to the reaction mixture, which was vortexed for 30 s; the upper phase containing the proline was measured using a spectrophotometer at 520 nm with toluene as a blank. Proline content (μmol/g fresh weight) was quantified by the ninhydrin acid reagent method using L-proline as a standard.

### Determination of total soluble protein and antioxidant enzyme activities

Frozen leaf and root tissue (1 g) was homogenized with 10 mL 0.1 M potassium phosphate buffer (pH 7.0), containing 0.1 mM EDTA-Na_2_, 0.5 mM ascorbate and 1% PVPP (polyvinyl polypyrrolidone) in an ice bath. The homogenate was filtered and centrifuged at 28,710 × *g* and 4°C for 10 min. The supernatant was used for determination of protein content and antioxidant enzyme activity.

Soluble protein concentration was determined as described by Bradford using bovine serum albumin as a standard [31].

Superoxide dismutase (SOD) activity was determined according to Giannopolitis and Ries [32]. Twenty μL enzyme solution was mixed with 3 mL SOD reaction solution (pH 7.8, 1.5 mL phosphate buffer, 0.3 mL 750 mol L^-1^ NBT, 0.3 mL 130 mmol L^-1^ Met, 0.3 mL 20 mol L^-1^ FD, 0.3 mL 100 mol L^-1^ EDTA-Na_2_, and 0.3 mL distilled water). Control and enzyme solutions were placed for 30 min in 4000 lux light. The blank was placed in the dark to set the zero, and the samples were measured at 560 nm.

Peroxidase (POD) activity was determined according to Hernández et al. [33]. In total, 20 μL enzyme solution was mixed with 3 mL POD reaction solution (1.4 μL guaiacol, 0.85 μL 30% H_2_O_2_, and 0.1 mol L^-1^ (pH 6.0) phosphate buffer). The absorbance values were recorded once every 30 s at 470 nm.

The guaiacol peroxidase (GPX) assay was performed using the method described by Egley et al. [34].

Catalase (CAT) activity was assayed as a decrease in absorbance at 260 nm for 1 min following the decomposition of H_2_O_2_ using the method described by Aebi [35]. The reaction mixture contained 50 mM phosphate buffer (pH 7.0) and 15 mM H_2_O_2_.

Ascorbate peroxidase (APX) activity was determined according to Nakano and Asada [36]. The assay mixture consisted of 0.5 mM ASA, 0.1 mM H_2_O_2_, 0.1 mM EDTA, 50 mM sodium phosphate buffer (pH 7.0), and 0.15 mL enzyme extract.

Glutathione reductase (GR) activity was assayed as described by Schaedle [37]. The oxidized GSH (GSSG)-dependent oxidation of NADPH was measured at 340 nm in a 1 mL reaction mixture containing 100 mM sodium phosphate buffer (pH 7.8), 0.5 mM GSSG, 50 μL extract, and 0.1 mM NADPH.

### Determination of ASA and GSH content

The ascorbic acid (ASA) content assay was performed using the method described by Kampfenkel et al. [38]. Glutathione (GSH) content was measured using the method described by Griffith [39].

All of the above assays were performed five times. Antioxidant enzyme activities are indicated as U/mg (enzyme activity unit number per mg protein).

### Statistical analysis

One-way analysis of variance was conducted with SPSS 21.0 software (SPSS Inc., Chicago, IL, USA). Duncan’s test method was employed for multiple comparisons and analysis of the differences; significance was set as *P* < 0.05. All data in the tables are average values of triplicate or more repetitions.

## Results

### Effect of *T*. *asperellum* on Na^+^, K^+^, and Ca^2+^ content in maize seedlings under saline–alkaline stress

**Fig 1:**
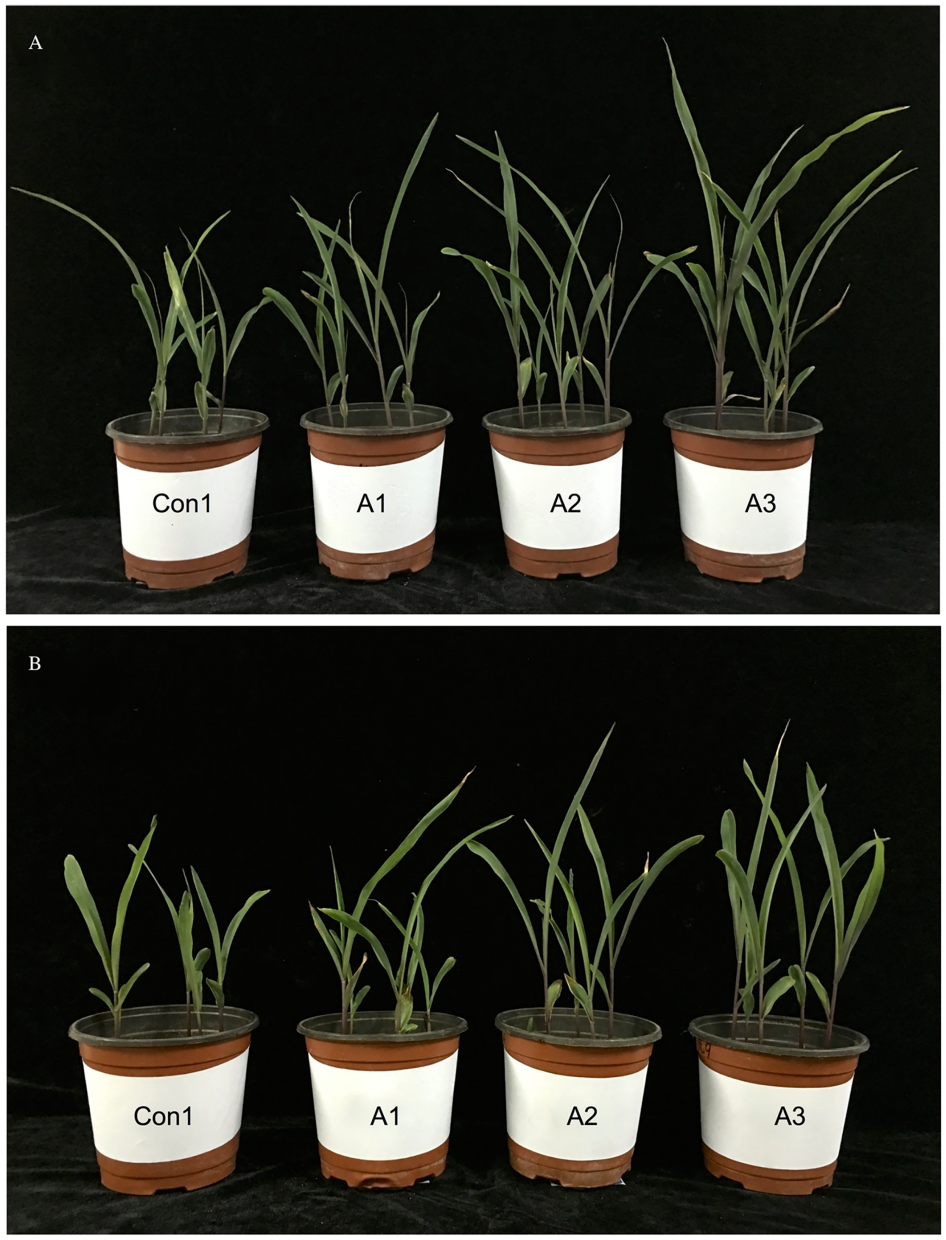
Maize seedlings before and after *T*. *asperellum* treatment. (A) The saline–alkaline-tolerant cultivar ‘Jiangyu 417’. (B) The saline–alkaline-sensitive cultivar ‘Xianyu 335’. Con1, A1, A2, and A3 indicate 0, 1 × 10^3^, 1 × 10^6^, and 1 × 10^9^ spores/L of fungal suspension, respectively. Photos were taken on 27^th^ d after *T*. *asperellum* application.

The leaves of 27-day-old seedlings in the *T*. *asperellum* treatment groups were larger than those in the Con1 control. In Con1, a saline–alkaline stress condition, the leaves showed an obvious loss of color and had turned yellow; by contrast, those treated with *T*. *asperellum* had green leaves. This characteristic gradually increased with the number of fungal spores in the suspension. The damage to maize leaves in Con1 was severe and the seedlings showed obvious wilting; this effect was ameliorated by treatment with *T*. *asperellum*. The seedlings treated with *T*. *asperellum* were notably larger than those in the Con1 treatment. Plant height increased with the increase in the concentration of fungal spores in the suspension; at the highest spore concentration (1 × 10^9^ spores/L), ‘XY335’ plants were 55.93% taller and ‘JY417’ plants were 39.42% taller than those in the Con1 treatment (Fig 1).

**Fig 2:**
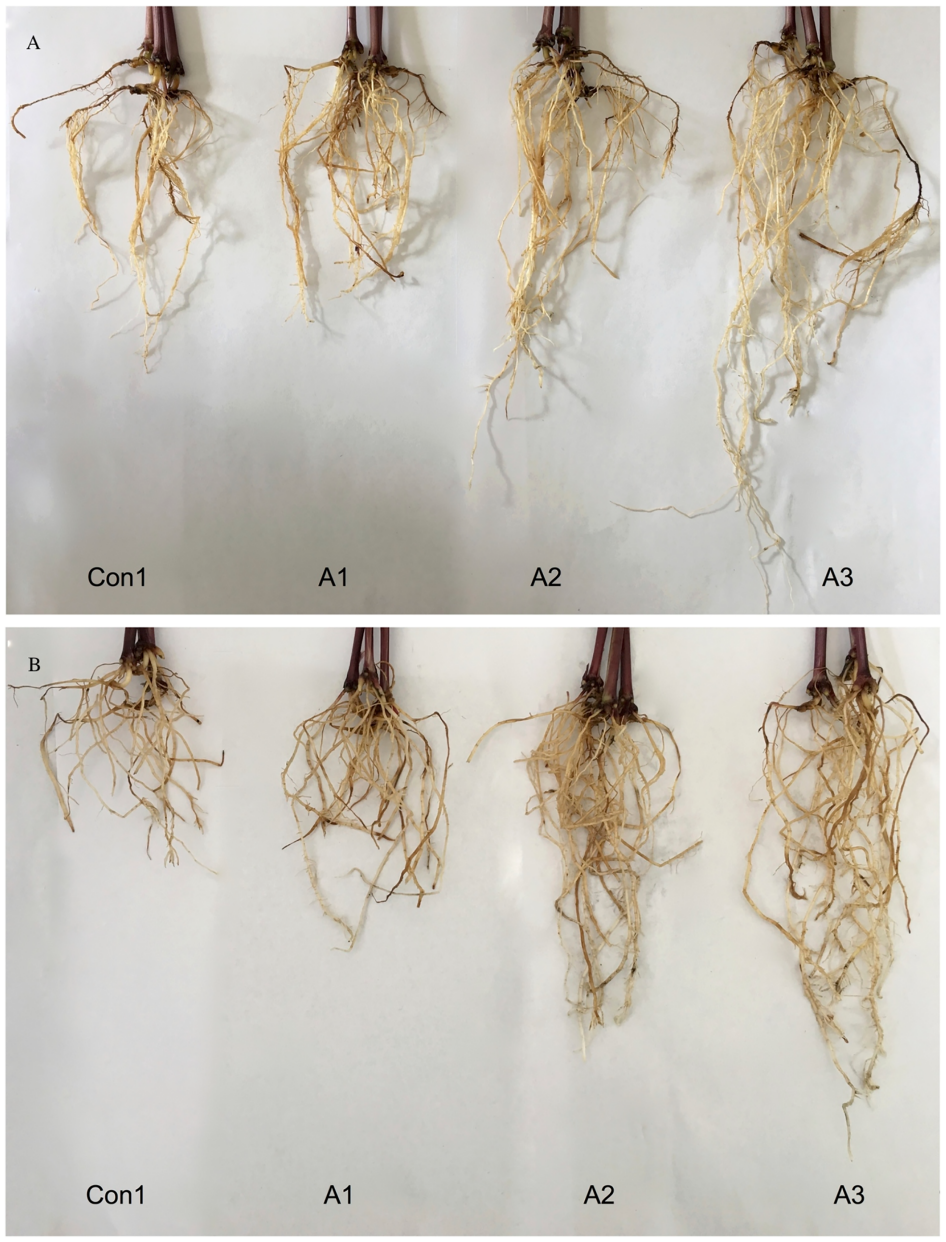
Maize roots in *T*. *asperellum* treated and untreated maize plants. (A) The saline–alkaline-tolerant cultivar ‘Jiangyu 417’. (B) The saline–alkaline-sensitive cultivar ‘Xianyu 335’. Con1, A1, A2, and A3 indicate 0, 1 × 10^3^, 1 × 10^6^, and 1 × 10^9^ spores/L fungal suspension, respectively. Photos were taken on 27^th^ d of *T*. *asperellum* treatment.

Root thickness in 27-day-old maize seedlings was larger after *Trichoderma* treatment compared to the Con1 group (Fig 2); root lengths were also greater after *T*. *asperellum* treatment, with the increase being related to the concentration of fungal suspension. Root dry weight increased by 57.97% in ‘XY335’ plants and by 43.53% in ‘JY417’ after *T*. *asperellum* treatment compared to the Con1 group. Moreover, the relative water content of roots increased by 8.98%, in ‘XY335’ plants and by 8.91%, in ‘JY417’ plants compared to Con1 plants. Our analysis of root characteristics indicated that *T*. *asperellum* treatments suppressed the deleterious effects of saline–alkaline stress on root growth.

**Fig 3:**
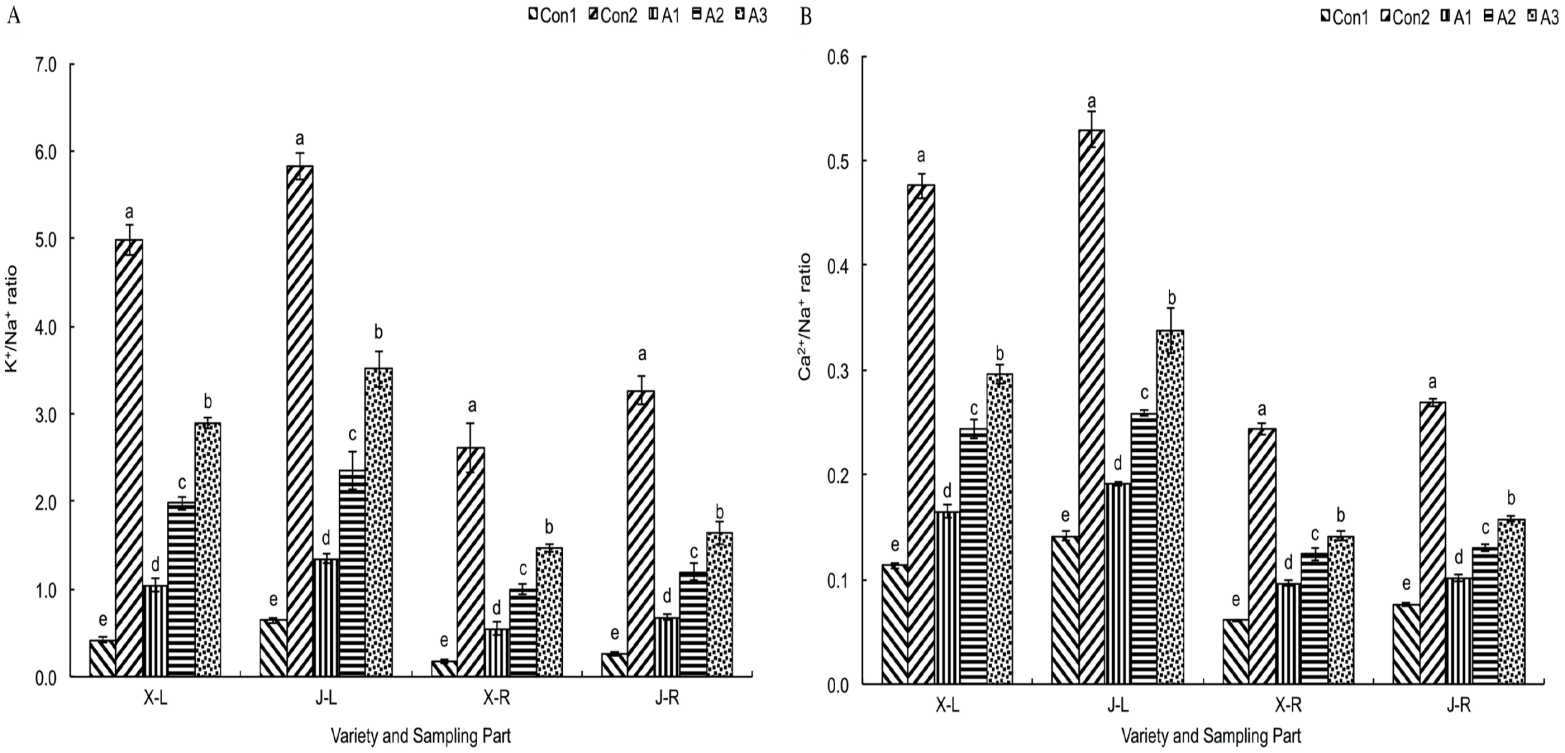
Influence of *Trichoderma* on the ratio of ion content in the leaves and roots of maize grown on saline–alkaline soil. (A) K^+^/Na^+^ ratio. (B) Ca^2+^/Na^+^ ratio. X–L: XY335 leaves, J–L: JY417 leaves, X–R: XY335 roots, J–R: JY417 roots. The determination of these parameters was done on the 27^th^ d after *T*. *asperellum* application. Note: Different lowercase letters in the figure indicate the significant differences (*P* < 0.05) between the different treatments. Vertical bars represent the standard error of means (±S.E).

The K^+^ and Ca^2+^ contents in the roots and leaves of ‘JY417’ and ‘XY335’ seedlings was notably lower in the Con1 group than in the control meadow soil (Con2), whereas the content of Na^+^ in Con1 was higher than in Con2. The content of K^++^ and Ca^2+^ in the leaves was higher than in the roots, whereas the content of Na^+^ in the leaves was lower than in the roots (Table 2). The K^+^-to-Na^+^ and Ca^2+^-to-Na^+^ ratios in the leaves and roots were much lower in the Con1 treatment. In the *T*. *asperellum* treatments, K^+^ and Ca^2+^ contents in leaves and roots of ‘JY417’ and ‘XY335’ plants increased in relation to the concentration of the fungal suspension. *T*. *asperellum* treatments produced a higher increase in K^+^ and Ca^2+^ contents in roots compared to leaves, and a clearly higher increase was observed in the treatment with 1 × 10^9^ spores/L. Moreover, treatment with *T*. *asperellum* lowered the content of Na^+^ in the leaves and roots of ‘JY417’ and ‘XY335’ plants; the content of Na^+^ in the roots was higher than in the leaves. The K^+^-to-Na^+^ and Ca^2+^-to-Na^+^ ratios in the leaves and roots of JY417 and XY335 were increased in the *T*. *asperellum* treatment groups (Fig 3A and 3B).

**Table 2:** Influence of *T*. *asperellum* on the ion content in the leaves and roots of maize grown on saline–alkaline soil.

Treatment^a^		XY335			JY417		
		Na^+^content^b^	K^+^content	Ca^2+^ content	Na^+^content	K^+^content	Ca^2+^ content
Leaf	Con1	28.128±0.232a^c^	11.759±0.799e	3.188±0.054e	24.179±0.421a	15.649±0.612e	3.421±0.058e
	Con2	8.857±0.194e	44.094±1.755a	4.212±0.017a	8.201±0.210e	47.727±2.439a	4.342±0.035a
	A1	21.630±0.947b	22.537±2.318d	3.556±0.054d	19.071±0.111b	25.642±0.806d	3.645±0.063d
	A2	15.274±0.378c	30.265±1.524c	3.735±0.055c	14.738±0.144c	34.675±2.979c	3.806±0.050c
	A3	13.007±0.304d	37.749±1.532b	3.850±0.034b	11.844±0.631d	41.548±0.360b	3.990±0.042b
Root	Con1	36.605±0.279a	6.631±1.009d	2.233±0.039d	31.868±0.456a	8.394±0.759e	2.436±0.025e
	Con2	12.962±0.273e	33.925±4.445a	3.148±0.010a	11.917±0.228e	38.938±1.369a	3.206±0.037a
	A1	28.080±0.342b	15.324±2.185c	2.698±0.092c	26.811±0.063b	18.279±1.066d	2.711±0.078d
	A2	23.014±0.440c	22.986±1.492b	2.847±0.084b	22.318±0.282c	26.599±2.446c	2.900±0.033c
	A3	20.747±0.231d	30.516±1.363a	2.933±0.084b	19.118±0.407d	33.293±1.919b	3.016±0.087b

Note:

^a^ Con1, A1, A2, and A3 indicate 0, 1 × 10^3^, 1 × 10^6^, and 1 × 10^9^ spores/L suspension, respectively; Con2 is the meadow soil control.

^b^ Ion content was measured on the 27^th^ d after *T*. *asperellum* application.

^c^ According to the Duncan’s test, different lowercase letters in the Table indicate the significance of difference (*P* < 0.05) between the different treatments, and the numerical value is the mean value of triplicate repeats ± SE

### Effect of *T*. *asperellum* treatment on the accumulation of ROS and on the oxidation parameters in maize seedlings under saline–alkaline stress

**Fig 4:**
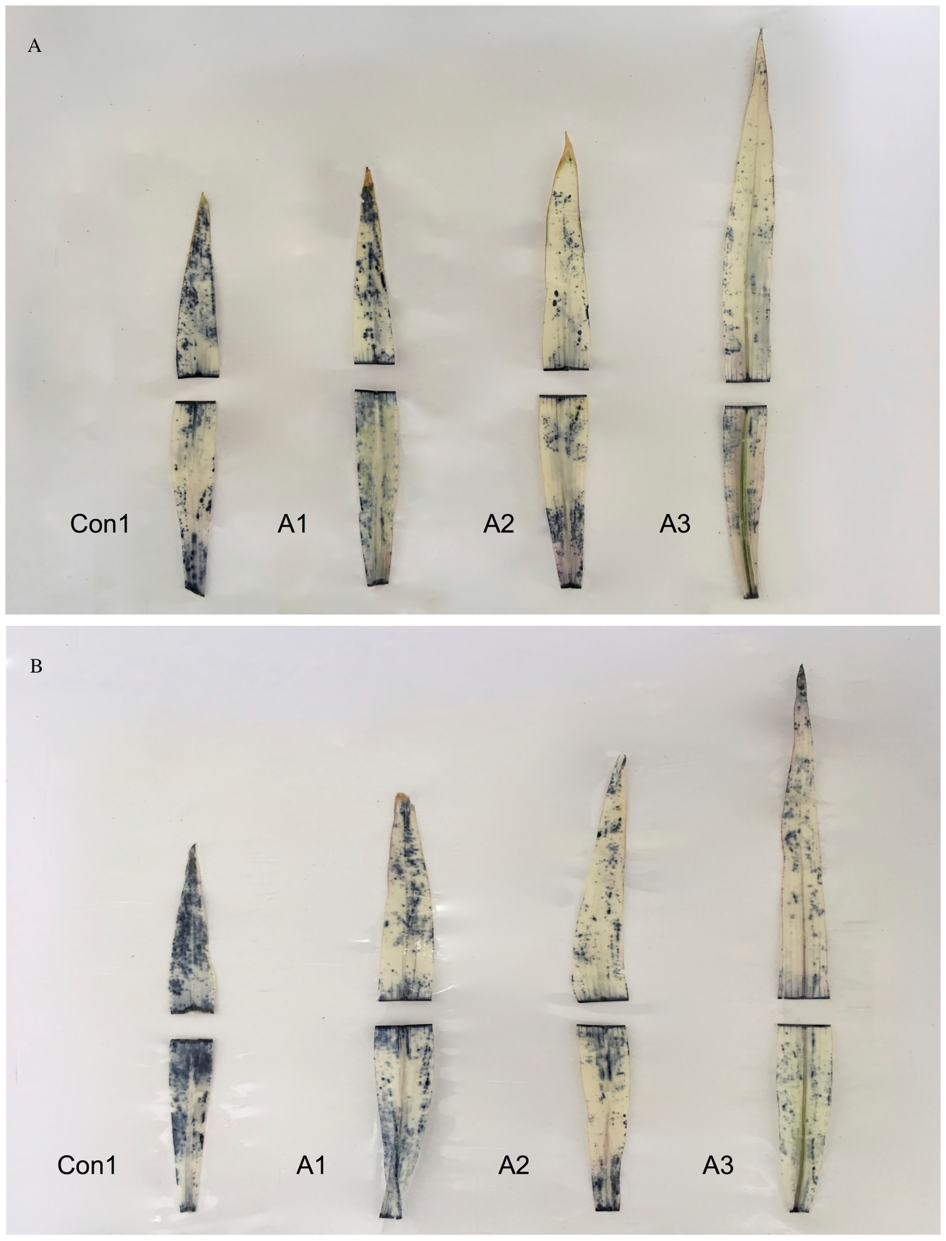
Nitroblue tetrazolium (NBT) staining of maize leaves. (A) Leaves from 27-d-old JY417. (B) Leaves from 27-d-old XY335. Con1, A1, A2, and A3 indicate 0, 1 × 10^3^, 1 × 10^6^, and 1 × 10^9^ spores/L of the fungal suspension, respectively, infiltrated with NBT. The staining was performed on the 3^rd^ leaf of the maize seedlings; the leaves were cut into two segments because of the difficulty in staining longer leaves.

The levels of reactive oxygen species in plants under stress were assessed using NBT staining. Treatment with *T*. *asperellum* resulted in a decrease in the intensity of the blue staining of leaves compared to the Con1 group. The reduction in intensity of staining was related to the concentration of the fungal spore solution. Thus, *T*. *asperellum* caused a dose-related decline in O_2_^-^ content. Leaves of ‘XY335’ plants stained more intensely than those of ‘JY417’ plants indicating that the two cultivars showed differences in their degrees of saline–alkaline stress damage (Fig 4).

**Fig 5:**
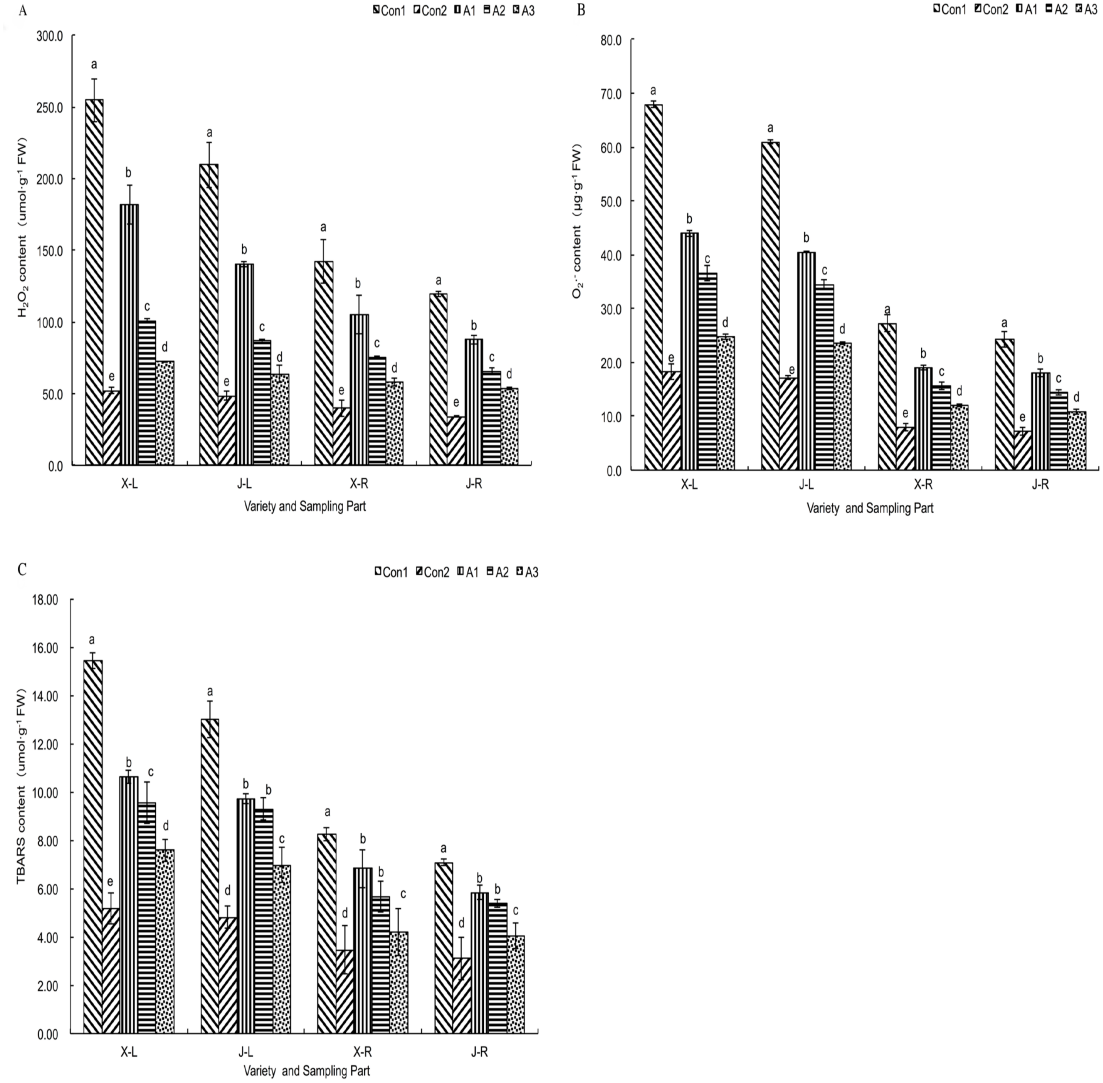
Influence of *T*. *asperellum* on the accumulation of ROS and on the oxidation parameters in the leaves and roots of maize seedlings in saline–alkaline soil. (A) H_2_O_2_ content. (B) O_2_^-^ content; (C) TBARS content, X–L: XY335 leaves, J–L: JY417 leaves, X–R: XY335 roots, J–R: JY417 roots. The determination of these parameters was done on the 27^th^ d after *T*. *asperellum* application. Note: Different lowercase letters in the figure indicate the significant differences (*P* < 0.05) between the different treatments. Vertical bars represent the standard error of means (± S.E.).

The leaves and roots of plants in the Con1 group had higher levels of TBARS and H_2_O_2_, as well as an increased rate of O_2_^-^ generation, compared to those grown in meadow soil (Con2). The levels of all three parameters were higher in leaves than in roots in all treatments. In the *T*. *asperellum* treatment groups, the accumulation of H_2_O_2_ and O_2_^-^ in leaves and roots was lower than in the Con1 groups (Fig 5A and 5B); peroxidation induced by saline-alkaline stress was reduced and, thus, TBARS accumulation was also reduced (Fig 5C). The greatest effect was observed at the highest concentration of fungal spore treatment (1 × 10^9^ spores/L). A similar pattern of response was seen in the two cultivars tested here. The decline in H_2_O_2_ content and the increase in O_2_^-^ generation were higher in leaves than in roots.

### Effect of *T*. *asperellum* treatment on soluble osmolytes in maize seedlings under saline–alkaline stress

**Fig 6:**
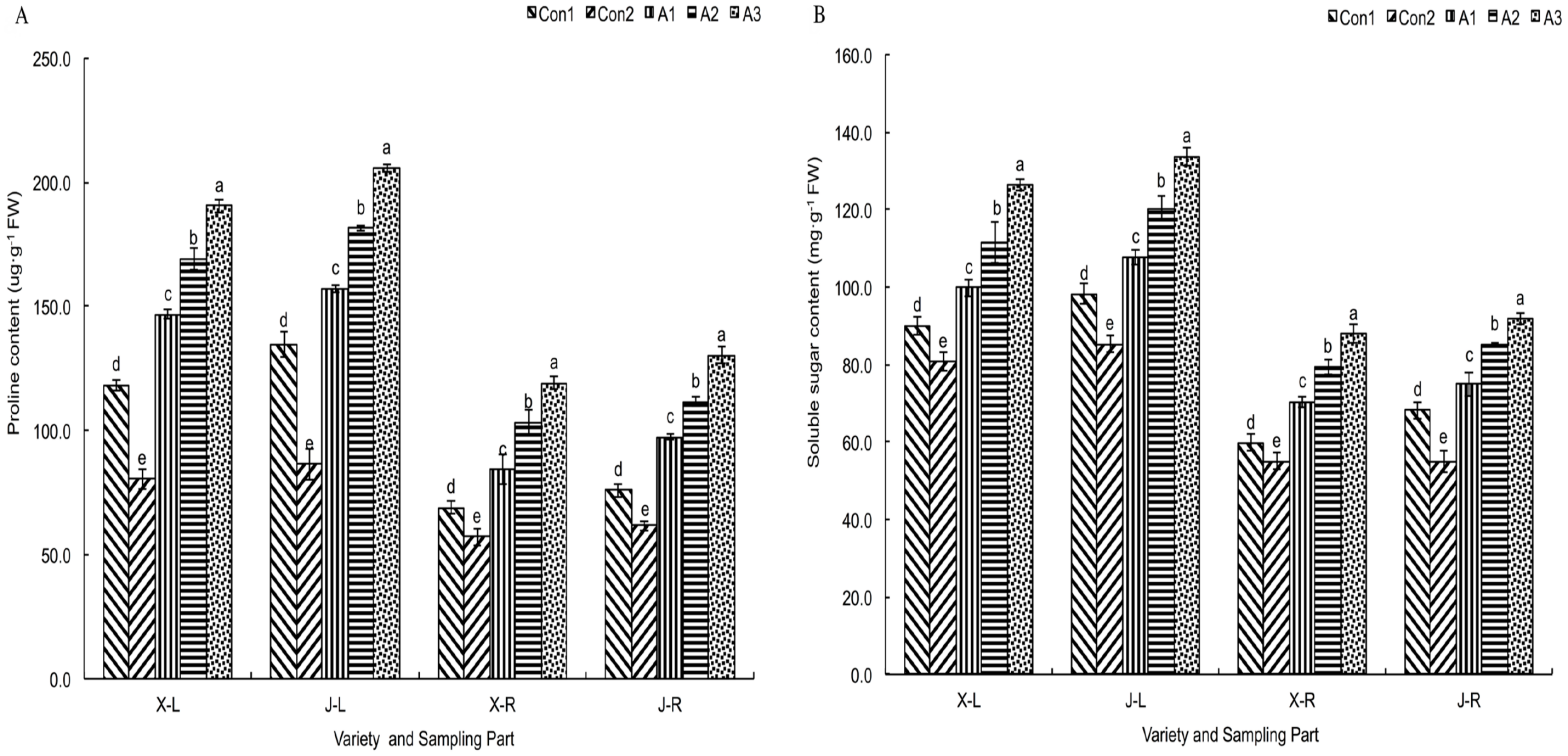
Influence of *T*. *asperellum* on the content of osmolytes in the leaves and roots of maize seedlings in saline–alkaline soil. (A) Proline content. (B) Soluble sugar content, X–L: XY335 leaves, J–L: JY417 leaves, X–R: XY335 roots, J–R: JY417 roots. The determination of these parameters was done on the 27^th^ d after *T*. *asperellum* application. Note: Different lowercase letters in the figure indicate the significant differences (*P* < 0.05) between the different treatments. Vertical bars represent the standard error of means (±S.E).

Osmotic adjustment is an important aspect of the development of stress resistance in crops. During osmotic adjustment, the cellular changes in the contents of soluble sugars and proline play an important role in the adjustment of osmotic pressure under adverse conditions, preventing the dehydration of cells. We found that the soluble sugar and proline contents of leaves and roots were higher in Con1 plants than in Con2 plants (Fig 6). In plants treated with *T*. *asperellum*, the soluble sugar and proline contents of the leaves and roots increased compared to Con1 plants. These increases were related to the concentration of *T*. *asperellum*. The effect of the treatment with the 1 × 10^9^ spores/L suspension was greater than that of other treatments, and the proline contents in the leaves and roots of ‘XY335’ and ‘JY417’ increased by 60.8 and 52.9%, and by 72.7 and 71.5%, respectively; soluble sugar contents increased by 40.3 and 36.2%, and by 47.2 and 34.5%, respectively, in the two cultivars. These results showed a larger increase in the roots than in the leaves, and also indicated that proline was more effective than soluble sugars in alleviating the osmotic stress.

### Effect of *T*. *asperellum* on the antioxidant enzyme activities of maize seedlings under saline–alkaline stress

**Fig 7:**
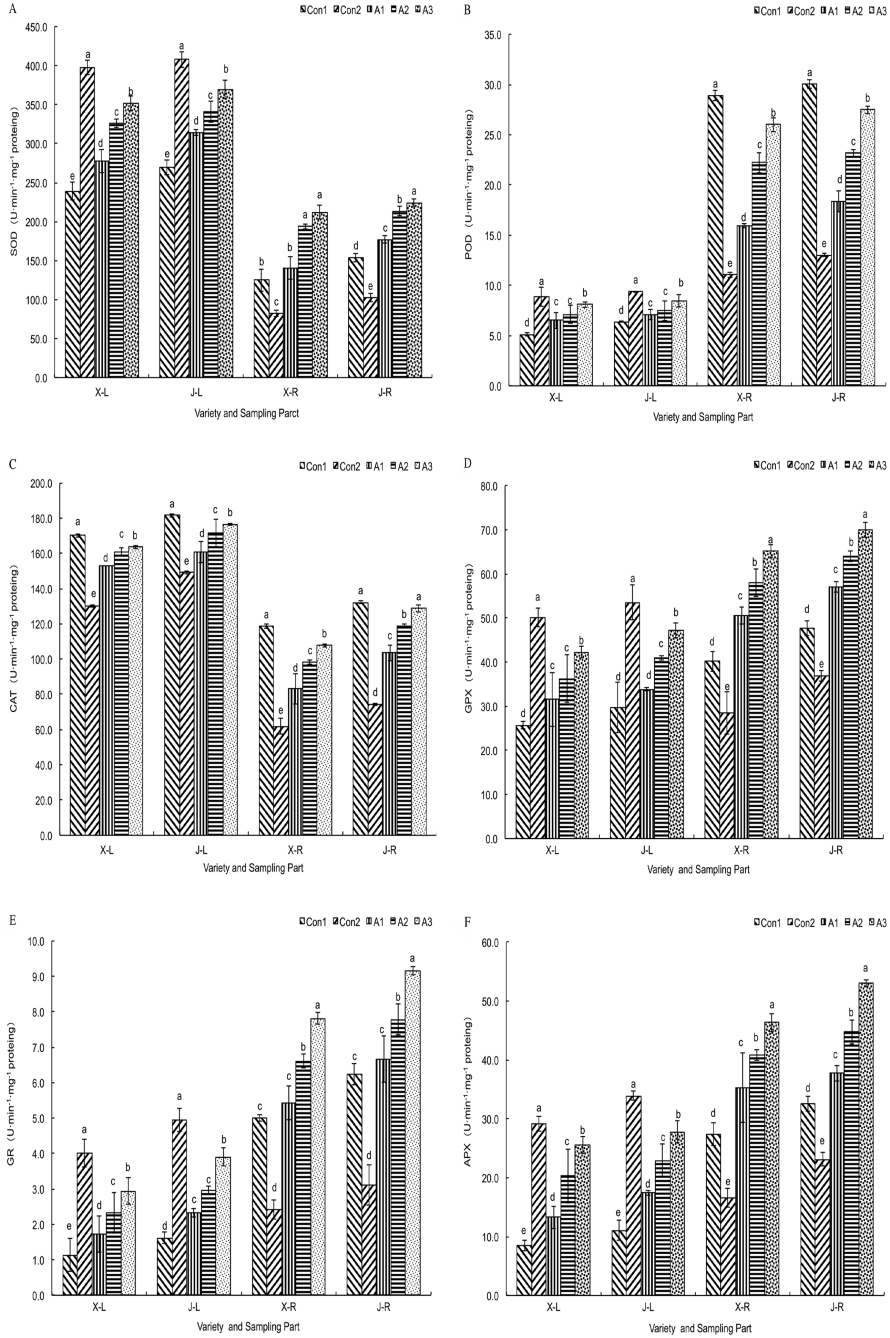
Influence of *T*. *asperellum* on the antioxidant enzyme activities in the leaves and roots of maize seedlings in saline–alkaline soil. (A) SOD activity. (B) POD activity. (C) CAT activity. (D) GPX activity. (E) GR activity. (F) APX activity. X–L: XY335 leaves, J–L: JY417 leaves, X–R: XY335 roots, J–R: JY417 roots. The determination of these parameters was done on the 27^th^ d after *T*. *asperellum* application. Note: Different lowercase letters in the figure indicate the significant differences (*P* < 0.05) between the different treatments. Vertical bars represent the standard error of means (±S.E).

The activities of SOD, APX, GPX, GR, and POD in leaves in the Con1 group were lower (*P* < 0.05) than those in plants grown in the Con2 treatment (Fig 7). However, the response of CAT activity was different; in the Con1 group, CAT activity in leaves increased and was higher than in other treatments. The effects of the stress on antioxidant enzyme activities in the roots in all the treatments differed from those in leaves. The activities of SOD, POD, APX, GPX, GR, and CAT in roots exhibited a tendency to increase under saline–alkaline stress, and were higher than those in the Con2 group. Enzymatic activities in plants of the two cultivars and in the different organs showed marked differences. In the ‘JY417’ cultivar, enzymatic activities were higher than in the ‘XY335’ cultivar. The activities of SOD and CAT were higher in leaves than in roots in all the treatments; GR, GPX, APX, and POD activities were lower in leaves than in roots. In the *T*. *asperellum* treatments, SOD, APX, GPX, and GR activities in both leaves and roots increased, and this increase was related to the spore concentration in the fungal suspension. The difference between the lowest spore concentration and the Con1 treatment was small; however, all the enzymes showed higher activities in the two higher spore concentrations. CAT activity in leaves and roots was lower in plants from the fungal suspension treatments compared to the Con1 group. POD activity in leaves and roots showed a different response with an increase in activity of POD in leaves with increasing fungal spore concentration; POD activity was higher in the fungal spore treatments than in the Con1 group, whereas that in roots was lower than in the Con1 group.

### Influence of *T*. *asperellum* on the content of non-enzymatic antioxidants in maize seedlings under saline–alkaline stress

As is evident from Tables 3 and 4, the ratios of non-enzymatic antioxidants (ASA-to-DHA and GSH-to-GSSG) in the leaves and roots of both cultivars in the Con1 treatment were lower than those in the Con2 group (*P* < 0.05). The reduction in the ‘XY335’ cultivar was higher than that in the ‘JY417’ cultivar. Moreover, a greater reduction was observed in the leaves the Con1 treatment group than in the roots. Total ascorbic acid and total glutathione content in the leaves and roots increased in the Con1 group compared to the Con2 group. The total content of ascorbic acid in leaves in the different *T*. *asperellum* treatments was higher than in the roots whereas the content of total glutathione in the leaves was lower than in the roots (Table 3). In addition, the reduction in ASA-to-DHA ratio in the leaves and roots was higher than that of GSH-to-GSSG.

**Table 3:** Effect of *T*. *asperellum* on the total ascorbic acid content and the ASA/DHA ratio in the maize leaves and roots on saline–alkaline soil.

Treatment^a^		XY335		JY417	
		ASA+DHA content^b^ (μmol·g^-1^ FW)	ASA/DHA content	ASA+DHA content(μmol·g^-1^ FW)	ASA/DHA content
Leaf	Con1	6.46±0.072d^c^	0.35±0.012e	7.19±0.062d	0.43±0.008d
	Con2	5.35±0.101e	3.15±0.041a	5.90±0.038e	3.35±0.233a
	A1	7.22±0.142c	0.51±0.033d	8.82±0.172c	0.71±0.036d
	A2	10.24±0.143b	0.85±0.010c	12.75±0.126b	0.98±0.017c
	A3	15.51±0.220a	1.46±0.035b	17.06±0.359a	1.62±0.017b
Root	Con1	4.87±0.069d	3.28±0.148d	5.63±0.091d	4.54±0.406e
	Con2	3.88±0.051e	13.80±0.666a	4.83±0.088e	17.94±0.701a
	A1	6.05±0.038c	4.70±0.532c	6.83±0.122c	5.81±0.501d
	A2	6.94±0.062b	6.29±0.725c	8.03±0.165b	7.38±0.750c
	A3	11.62±0.109a	11.84±0.139b	13.11±0.116a	12.80±0.907b

Note:

^a^ Con1, A1, A2, and A3 indicate 0, 1 × 10^3^, 1 × 10^6^, and 1 × 10^9^ spores/L suspension, respectively; Con2 is the meadow soil control.

^b^ Total ascorbic acid content was measured on the 27^th^ d after *T*. *asperellum* application.

^c^ According to the Duncan’s test, different lowercase letters in the Table indicate the significant differences (*P* < 0.05) between the different treatments, and the numerical value is the mean value of triplicate repeats ± SE

**Table 4:** Effect of *T*. *asperellum* on the total glutathione content and the GSH/GSSG ratio in the maize leaves and roots on saline–alkaline soil.

Treatment^a^		XY335		JY417	
		GSSG+GSH content^b^(μmol·g^-1^ FW)	GSH/GSSG content	GSSG+GSH content(μmol·g^-1^ FW)	GSH/GSSG content
Leaf	Con1	534.84±1.671d^c^	2.05±0.044d	543.44±6.204d	2.20±0.069d
	Con2	509.96±6.323e	5.72±0.052a	517.78±6.776e	5.94±0.028a
	A1	589.02±6.210c	4.42±0.200c	598.14±2.838c	4.60±0.169c
	A2	620.50±10.406b	4.92±0.051b	634.13±7.268b	5.27±0.088b
	A3	654.93±9.373a	5.61±0.060a	664.74±6.075a	5.79±0.066a
Root	Con1	585.98±1.962d	3.16±0.120e	601.11±7.718d	3.58±0.196e
	Con2	550.34±5.161e	7.55±0.113a	568.91±7.358e	8.04±0.310a
	A1	640.15±6.088c	5.36±0.195d	649.27±2.161c	5.66±0.066d
	A2	671.63±9.811b	6.20±0.179c	685.27±7.484b	6.56±0.205c
	A3	706.06±10.395a	7.08±0.183b	715.87±5.308a	7.49±0.107b

Note:

^a^ Con1, A1, A2, and A3 indicate 0, 1 × 10^3^, 1 × 10^6^, and 1 × 10^9^ spores/L suspension, respectively; Con2 is the meadow soil control.

^b^ Total glutathione content was measured on the 27^th^ d after *T*. *asperellum* application.

^c^ According to the Duncan’s test, different lowercase letters in the Table indicate the significant differences (*P* < 0.05) between the different treatments, and the numerical value is the mean value of triplicate repeats ± SE

Total ascorbic acid and glutathione contents as well as the ASA-to-DHA and GSH-to-GSSG ratios in leaves and roots of both cultivars increased with increasing concentration of fungal spore suspension. At the highest fungal suspension concentration, the leaves showed a 2.4-fold and 1.2-fold increase in ascorbic acid and glutathione contents, respectively, in ‘XY335’ plants, and 2.4-fold and 1.2–fold increase, respectively, in ‘JY417’ plants. Ascorbic acid and glutathione contents in roots increased 2.4-fold and 1.2-fold, respectively, in ‘XY335’ plants, and 2.3-fold and 1.2-fold, respectively, in ‘JY417’ plants. The increase was higher in leaves than in roots, and was also higher in the saline–alkaline sensitive cultivar. The ASA-to-DHA and GSH-to-GSSG ratios in leaves increased 4.1-fold and 2.7-fold, respectively, in ‘XY335’ plants, and 3.8-fold and 2.6-fold, respectively, in ‘JY417’ plants; the ratios increased by 3.6-fold and 2.2-fold, respectively, in roots of ‘XY335’ plants, and 3.0-fold and 2.1-fold in ‘JY417’ plants. Thus, we conclude that application of *T*. *asperellum* had a positive effect on relieving saline–alkaline stress and could increase total ASA and GSH contents as well as the ratios of ASA-to-DHA and GSH-to-GSSG in leaves. The treatment may effectively relieve the saline–alkaline damage by ASA-GSH circulation, improve the redox potential in cells, and thus, enhance the antioxidant ability of cells and maintain the metabolic balance of active oxygen in cells.

## Discussion and conclusion

Previous studies have used *Trichoderma* as a biocontrol treatment for crop plants. For example, Shoresh et al. used *Trichoderma* at a 1–2×10^9^ spore concentration to treat maize seedlings and studied the effects using proteomics methods [40]. Guler et al. used a 1×10^7^ concentration to improve the oxidation resistance of maize seedlings under drought stress [19]. Zhang et al. applied a concentration of 1×10^8^ to improve salt resistance in wheat through improving the antioxidant system [20]. Pandey et al. used a 1×10^7^ concentration of *Trichoderma* to alter the physiological and molecular mechanisms of drought resistance in rice [21]. Kumar et al. applied a 1×10^8^ concentration of *Trichoderma* to improve salt resistance in maize [22]. Brotman et al. used a concentration of 1×10^6^ to investigate the effects on oxidation resistance in cucumber under salt stress [13]. Singh et al. applied a 2×10^7^ concentration of *Trichoderma* to increase antioxidant activity in soybean [23]. In the present study, following a preliminary test, we chose to apply a high concentration of *Trichoderma* spores of 1×10^9^ spores/L.

Under saline–alkaline stress, harmful ions including Na^+^ enter the plant in large amounts and accumulate there. These ions can disrupt water balance and inflict damage to the plant. The plant roots can selectively absorb and restrict the entry of Na^+^, and also help in its excretion and compartmentation. Roots maintain the ion balance and reduce the influence of salt damage. Ions such as K^+^ and Ca^2+^ play important roles in salt tolerance in plants [41]. The results of this study indicated that saline–alkaline stress severely damaged the ionic equilibrium in leaves and roots of ‘XY335’ and ‘JY417’ seedlings. Treatment with a *T*. *asperellum* suspension increased the content of K^+^ and Ca^2+^ in the leaves and roots of the seedlings and decreased the Na^+^ content; the ratios of K^+^-to-Na^+^ and Ca^2+^-to- Na^+^ were increased by *T*. *asperellum* treatments. Thus, application of *T*. *asperellum* increased the ion content, enhanced ionic equilibrium, and alleviated the damage due to ion toxicity and thereby improved the saline–alkaline tolerance of the plants. The results of this study are in agreement with those reported previously [17, 18].

Our analyses also showed that, in comparison to meadow soil (Con2), the leaves and roots of ‘XY335’ and ‘JY417’ plants under saline-alkaline stress (Con1) had altered levels of proline and soluble sugar. In Con1 plants, these accumulated in large quantities and altered the osmotic equilibrium of cells; they also act as protective agents to counter the effects of ROS. This result is consistent with the report of Wang et al. who concluded that saline–alkaline stress stimulated the accumulation of proline and enhanced saline–alkaline resistance [42]. Treatment with *T*. *asperellum* suspensions caused an increase in proline and soluble sugar contents in leaves and roots of both ‘XY335’ and ‘JY417’. The average content in leaves was higher than in roots suggesting that the leaves might accumulate more osmolytes to aid transport of water from the roots. There were certain differences between the permeation effects of the osmolytes between the two cultivars and among the different tissues. Generally speaking, the effect of proline was more obvious for the saline–alkaline-sensitive cultivars. This might be a response to *T*. *asperellum*-mediated induction of phytohormone production [43, 44].

Saline–alkaline stress can result in the accumulation of ROS in cells and damage the dynamic equilibrium of ROS that are present in plants under normal conditions [45]. The stress conditions also cause an increase in the activities of antioxidant enzymes and in the content of reducing substances in plants. Saline-alkaline stress therefore initiated the activity of antioxidant defense systems to control the toxic action of ROS. Previous studies have shown that saline–alkaline stress increases the activities of some antioxidant enzymes [46, 47], but not of all such enzymes [48]. The differences in response indicate that different types of antioxidant enzyme have specific functions and that they have a clear division of labor for eliminating ROS. Therefore, efficient antioxidant activity does not necessarily mean up-regulation of the activities of all the antioxidant enzymes, and vice versa [49]. In the present study, we found that saline–alkaline stress caused different variations in antioxidant enzyme activities in leaves and roots of ‘XY335’ and ‘JY417’ cultivars. These differences might be due to the generation of ROS by saline–alkaline stress exceeding the capacity for their removal by the antioxidant defense system in the leaves. For example, if the capacity of APX, GPX, and GR to remove H_2_O_2_ is overwhelmed, then the leaves might suffer severe damage [50, 51]. ROS content in the roots was also found here to increase, but this increase was less than observed in leaves. However, the increase found here in CAT activities in leaves and roots might have resulted from the accumulation of ROS. However, previous studies indicate that the affinity of CAT toward H_2_O_2_ is weak [52] and, therefore, it is not able to lower the concentration of H_2_O_2_ to physiologically acceptable levels. As a result, the occurrence of high CAT activity is an indication that the plants are under oxidative stress. Our results here support this conclusion.

The GSH-to-GSSG and ASA-to-DHA ratios in leaves were lower than those in roots; these ratio differences might be due to variations in enzymatic activities in leaves and roots (Fig 7). First, the activities of GR and APX in leaves were lower than in the roots. Second, the content of GSSG+GSH in leaves was lower than in the roots (Table 4). These observations are consistent with the high GR activity in roots, which guarantees successful ASA-GSH circulation in the roots that rapidly converts GSSG and DHA to GSH and ASA, respectively. Third, the content of ASA+DHA in the leaves was higher than in the roots (Table 3). This might mean that ASA in leaves actively participates in eliminating ROS.

*T*. *asperellum* treatment led to an increase in the activities of SOD, APX, GPX, and GR in leaves and roots of ‘XY335’ and ‘JY417’ plants. The effect of the treatment was greater in ‘XY335’ than in ‘JY417’. Total ascorbic acid and glutathione contents, as well as the ratios of GSH-to-GSSG and ASA-to-DHA, were increased in leaves and roots. Moreover, *T*. *asperellum* reduced ROS in leaves and roots and, thus, reduced the accumulation of TBARS. The reduction in ROS and TBARS in leaves was greater than that in roots. These results indicate that *T*. *asperellum* can effectively improve resistance to oxidation in the leaves and roots of ‘XY335’ and ‘JY417’ plants under saline–alkaline stress. Moreover, *T*. *asperellum* treatment increased the ascorbic acid and glutathione contents in the plants and reduced the generation of ROS, thus reducing the damage caused by oxidative stress on the cell membrane system of the plants. The alleviation of stress was greater in leaves than in roots. These results are consistent with those of previous studies, which showed that *T*. *asperellum* treatment of cucumber under NaCl stress increased antioxidant enzyme activities [13]. Here, we found that CAT activity was reduced following the reduction in H_2_O_2_ content in leaves and roots after treatment with *T*. *asperellum*.

In a previous study on *Trichoderma*-mediated induction of drought-resistant mechanisms in rice, we found that the effect increased with the concentration of fungal spores applied [21]. In the present study of saline-alkaline soil stress the *Trichoderma*-induced remission effect in the maize seedlings increased with fungal spore concentration. Of the three fungal treatments tested here, the 1×10^9^ spores/L was the most effective. This conclusion is similar to that reported in previous studies [21].

Previous studies have reported that *Trichoderma* can induce plant resistance to abiotic stresses, including drought, saline, and alkaline conditions through improving antioxidant defense capabilities in the plants to reduce the damage caused by the stress [19, 20, 53, 54]. However, the complex nature of natural saline–alkaline soils makes it difficult to mimic these conditions in experimental systems. Moreover, different mechanisms of salt alleviation and growth promotion may be present in different plant cultivars. In this study, *T*. *asperellum* treatment showed alleviation effects toward cultivars with different genotypes under the stress of a natural saline–alkaline soil. Possibly, a drop irrigation method might be used to apply *T*. *asperellum* to a crop in the field. Potentially, the use of *T*. *asperellum* application could be used at a large scale to improve crop yields from saline–alkaline land.

In conclusion, treatment with *T*. *asperellum* reduced ROS levels by increasing the activities of antioxidant enzymes in maize seedlings grown under saline–alkaline stress. Thus, the treatment alleviated peroxidation damage of membrane lipids. In addition, the treatment was helpful in maintaining the balance among the major mineral elements by increasing the content of K^+^ and Ca^2+^ in the roots and leaves. Moreover, the treatment also induced the accumulation of osmolytes in the plants, which helped to improve the water absorbing capacity of cells and, thus, enhanced the saline–alkaline resistance of the maize seedlings. We suggest that *T*. *asperellum* may be valuable for increasing resistance to saline–alkaline stress in maize.

## Supporting information

S1 File Supporting information data. This file contains data including ion content, TBARS content, H_2_O_2_ content, O_2_^-^ content, proline and soluble sugar, antioxidant enzyme activity, non-enzymatic antioxidants content.
(XLSX)
